# Modern Machine Learning Practices in Colorectal Surgery: A Scoping Review

**DOI:** 10.3390/jcm11092431

**Published:** 2022-04-26

**Authors:** Stephanie Taha-Mehlitz, Silvio Däster, Laura Bach, Vincent Ochs, Markus von Flüe, Daniel Steinemann, Anas Taha

**Affiliations:** 1 Clarunis, University Center for Gastrointestinal and Liver Diseases, St. Clara Hospital—University Hospital Basel, 4002 Basel, Switzerland; stephanie.taha@clarunis.ch (S.T.-M.); silvio.daester@clarunis.ch (S.D.); markus.vonfluee@clarunis.ch (M.v.F.); daniel.steinemann@clarunis.ch (D.S.); 2 Faculty of Medicine, Friedrich-Alexander University, 91054 Erlangen, Germany; laura.bach@fau.de; 3 Roche Innovation Center Basel, Department of Pharma Research & Early Development, 4070 Basel, Switzerland; vincent.ochs@unibas.ch; 4 Department of Biomedical Engineering, Faculty of Medicine, University of Basel, 4123 Allschwil, Switzerland

**Keywords:** machine learning, colorectal surgery, PubMed database, Google Scholar, Cochrane library

## Abstract

Objective: The use of machine learning (ML) has revolutionized every domain of medicine. Surgeons are now using ML models for disease detection and outcome prediction with high precision. ML-guided colorectal surgeries are more efficient than conventional surgical procedures. The primary aim of this paper is to provide an overview of the latest research on “ML in colorectal surgery”, with its viable applications. Methods: PubMed, Google Scholar, Medline, and Cochrane library were searched. Results: After screening, 27 articles out of 172 were eventually included. Among all of the reviewed articles, those found to fit the criteria for inclusion had exclusively focused on ML in colorectal surgery, with justified applications. We identified existing applications of ML in colorectal surgery. Additionally, we discuss the benefits, risks, and safety issues. Conclusions: A better, more sustainable, and more efficient method, with useful applications, for ML in surgery is possible if we and data scientists work together to address the drawbacks of the current approach. Potential problems related to patients’ perspectives also need to be resolved. The development of accurate technologies alone will not solve the problem of perceived unreliability from the patients’ end. Confidence can only be developed within society if more research with precise results is carried out.

## 1. Introduction

Machine learning (ML) and neural networks can be amalgamated to improve existing technologies and to create novel ones. ML involves computer systems simulating human intelligence processes. Potential applications of ML in cancer detection, surgery, and postoperative care have changed the diagnostic and outcome of colorectal cancer [1]. In modern surgical approaches, we can use ML systems to collect data from medical professionals performing surgery [2]. In combination with ML, the collected data allows surgical robots to develop reasoning, wherein the robots can undertake functions like decision-making and problem-solving.

ROBODOC systems, introduced in 1992, revolutionized the medical domain by introducing ML into medicine [3]. Recently, several researchers have utilized ML with numerous benefits, such as cost reduction and higher levels of patient satisfaction, especially when compared to conventional methods involving only human doctors. Researchers are already working on applications of ML at the preoperative stage, for instance, in the detection of existing ailments [4]. Using ML algorithms for colonic investigations has been a breakthrough [5]. Researchers are working to examine the future and further implementations of ML, particularly in the field of surgery. 

A persistent problem in this field is the lack of experiments and studies focusing on the future of ML. The increase of publications considering ML in medicine, in general, has also made it necessary to conduct this review. Moreover, there is a lack of research focusing on the applications of ML in colorectal surgery specifically.

The results of this review will be beneficial for fellow scholars to add to their knowledge on this topic. Students, for instance, could use this study as a reference in projects which are part of their curriculum. This collective information will also contribute to future research. In addition, the information acquired through this research will be not only beneficial for patients undergoing colorectal surgery but also for various medical practitioners. Both surgeons and patients will have access to more knowledge on the benefits of ML and its future in surgery. Furthermore, this will help clinicians to realize the various unexplored opportunities of ML and devise ways of maximizing them in the future. The developers of different ML systems may benefit from this research as well, as it may guide them to improve the development of techniques.

This study aims to summarize the current extent of ML in colorectal surgery and determine its future scope. To achieve this, the objectives were:

o To discuss the existing applications of ML in colorectal surgeries.
o To discuss the possibility of safe applications of ML in clinical environment.
o To examine the benefits of utilizing ML in the surgical domain and its future scope.

## 2. Methods

### 2.1. Search Strategy

We used various search engines, such as the PubMed database, Google Scholar, Medline and Cochrane library, to search for articles. We preferred and selected articles which appeared mutually in all the search engines we used. In addition, the articles which were relevant to our theme were given priority. After comparing the main results of the search engines, we found that the articles mentioned in this paper contained the most relevant information and had large overlaps (>95%) with the search engines we decide not to use.

Later, a screening of articles published between 2015 and 2021 was performed, as they included the most up-to-date information. The articles published before 2015 were not considered because they were more likely to include outdated information. The PRISMA approach for scoping reviews was then used to choose the articles for our final reference and, finally, to draft this study. 

The terms searched for were ‘colorectal surgery’ and ‘machine learning in surgery’, ‘the application of machine learning in surgery’, ‘machine learning in colorectal surgery’ and ‘the future of machine learning in colorectal surgery’. Any search using artificial intelligence (AI) as keyword would have provided ambiguous results of articles, as ML is a part of much broader field known as AI. Whereas the articles we referenced have brief discussions on AI, this topic was discussed by the authors to merely show the beautiful amalgamation of two different fields.

Furthermore, the Boolean operators were used to combine the terms to guarantee a vast search. In addition, we reviewed the references in the chosen studies manually. Three authors engaged in the search strategy individually. Discrepancies were discussed and solved by re-reviewing the article to establish a consensus decision. 

### 2.2. Selection of Criteria and Evidence Quality

Only English literature and full-text analyses were included in this review. To ensure the accuracy of the information, only peer-reviewed studies were included, as the quality of their results was considered reliable. With the assistance of a skilled librarian, the authors devised the search strategy, and the librarian who drafted the data sources also peer-reviewed the search strategy. The PRESS approach was used for the peer review to determine whether the search strategy matched the research topic [6]. We excluded studies that failed to address the research topic, and to avoid duplicates, only the latest publication of a research group was included. Among all data and information available in the precisely chosen articles, the most relevant data was the type of surgery. Since this study specifically focused on colorectal surgery, only information pertaining to it was selected. 

ML uses two types of techniques: supervised learning, which trains a model on known input and output data so that it can predict future outputs, and unsupervised learning, which finds hidden patterns or intrinsic structures in input data.

Supervised machine learning builds a model that makes predictions based on evidence in the presence of uncertainty. A supervised learning algorithm takes a known set of input data and known responses to the data (output) and trains a model to generate reasonable predictions for the response to new data. Supervised learning is used if you have known data for the output you are trying to predict.

Unsupervised learning finds hidden patterns or intrinsic structures in data. It is used to draw inferences from datasets consisting of input data without labelled responses. Both types of models can be used to detect patterns in surgery.

### 2.3. Data Extraction

A data charting form was established, including the study design, year of publication, and the theme discussed in the article. This helped the authors to categorize the reviews based on relevance. We assessed the reliability, usefulness, and authority of the studies to ensure quality evidence for this review. The extraction of information depended on discussions between the authors, including their perspective on ML in colorectal surgery. The types of studies that matched our eligibility criteria are summarized in Table 1. Furthermore, a count of included and excluded studies due to failure to meet the developed criteria was kept.

## 3. Results

### 3.1. Selection of Sources of Evidence

Selecting sources involved three steps in addition to the inclusion and exclusion criteria. The primary step was to identify the theme of the articles. This involved categorizing all articles sourced from the PubMed database, Google Scholar, Medline, and Cochrane library. This part also involved the recognition of the articles that underwent elimination before screening, either because of duplication or ineligibility. 

**Figure 1:**
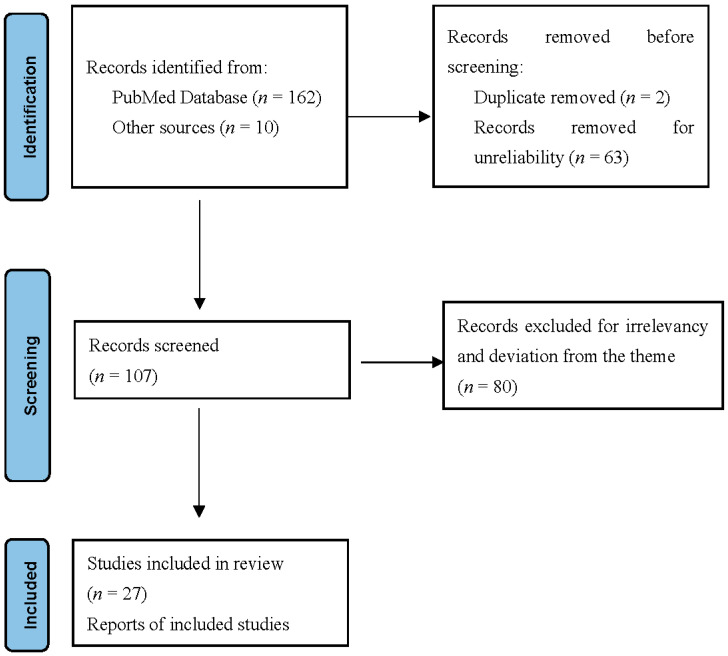
Identification of studies. Inclusion and exclusion criteria adopted for this research.

Further screening of the remaining articles was performed to identify those eligible for the study. The publications included in the research are shown in Figure 1.

### 3.2. Characteristics of Evidence Sources

A summary of the articles included in the study is presented in Table 1.

**Table 1:** Summary of included articles.

Authors	ML Algorithm	Study Design and Summary
Hashimoto, et al. [2]	-	Surgeons are the most significant enablers of ML adoption. Computer Vision and Natural Language Processing are popular subfields of AI used to derive insights. Review on ML in colorectal surgery.
Beyaz [3]	ROBODOC	ML in surgery began in 1992. The paper described the development of ML in surgery and the problems surgeons have had implementing ML in their practice.
Kitaguchi et al. [7]	convolutional neural network (CNN)	Automated colorectal surgery workflow recognition using a CNN to identify surgical stages and diagnosis of surgical actions with 82% accuracy.
Wang et al. [8]	FR-CNN	ML in colorectal surgery has received a lot of interest. Authors worked on ML-assisted pathological biopsy. CNN efficient in managing colorectal cancer.
Park et al. [9]	AI Real-time Analysis Microperfusion	To create an ML-based real-time analytic model for indocyanine green angiography during colorectal surgery. AIRAM model accuracy and consistency higher compared to traditional approaches.
Mitsala et al. [10]	CNN	Use of CNN in diagnosing, screening, and treating colorectal cancer. Performing analysis on medical images with the help of CNN.
Wang and Dong [11]	C-CAD (Conventional Computer Assisted Diagnosis)	C-CAD is effective in the detection of colorectal adenomas and cancer.
Merath et al. [12]	DT (Decision Tree)	DT algorithms predict the occurrence of complications after colorectal surgery. Complications were predicted in 13 of 17 instances.
Yamashita et al. [13]	MSINet Model (Microsatellite Instability)	The authors used ML in their approach by developing a deep learning model titled MSINet, which was found to be successful in forecasting MSI in colorectal cancer patients.
Echle et al. [14]	ShuffleNet	ShuffleNet is a deep learning system designed to detect MSI in patients with colorectal cancer. The study found this model to be accurate in predicting MSI in colorectal cancer.
Ahmad et al. [15]	CNN	CNN is now the most often utilised strategy in colorectal surgery, according to the authors. Many scholars have proposed using image magnification ML algorithms in clinical practice.
Skrede et al. [16]	Deep Learning (DL)	ML-based prognostic markers effectively classified colorectal cancer patients into two phases, allowing surgeons to choose appropriate treatment while avoiding overtreatment of low-risk patients.
Kudo et al. [17]	ANN (Artificial Neural Network)	ANN effectively recognized patients with T1 colorectal cancer with lymph node metastases to identify individuals who require additional surgery following endoscopic resection.
Yuan et al. [18]	Residual Networks + SVM (Support Vector Machine)	ResNet + SVM classifier can detect synchronous peritoneal carcinomatosis in colorectal cancer. The model gave 94% accuracy rate.
Ichimasa et al. [19]	Machine Learning (ML)	The use of ML in colorectal cancer surgery gives a successful prediction, lowering the need for additional procedures after endoscopic resection of T1 tumors.
Loftus et al. [20]	ML	The use of ML in colorectal cancer improves decision-making by augmenting informed consent and the choice to operate. ML-based electronic health also records algorithms.
Hildebrand et al. [21]	CNN	CNN predicts immunotherapy responses for cancer patients and detects microsatellite instability.
Luo et al. [22]	ML	ML-automated polyp detection system could increase polyp detection rate. Increasing the use of ML in colorectal surgery can minimize surgeon load while maintaining service efficiency.
Wang, Deng, and Wu [23]	CNN	ML models can also use magnetic resonance imaging (MRI) results as inputs. This has been proved effective in predicting the responses of various patients towards chemotherapy with an accuracy rate of 95%.
Chen et al. [24]	DL	Humans, unlike computers, cannot detect algorithmic patterns. However, most ML methods need complicated processing, making data extraction tedious. Although, DL and alternative learning strategies for retrospective real-world clinical data have proved to be a boon.
Hardy et al. [25]	CNN	CNN models are efficienct in diagnosing, screening, and treating colorectal cancer. This suggests that researchers will likely improve colorectal cancer detection technologies to aid in successful treatment.
Shung and Byrne [26]	CNN	CNN have enhanced the quality of colonoscopy processes and also helped in cancer screening.
Gao et al. [27]	FR-CNN	FR-CNN allows to detect malignancies and recommend treatment options which is effective in diagnosing colorectal cancer.
McKendrick et al. [28]	ML	ML encourages the development of mixed tech but more ML algorithms will need to be developed and improved.
Dias, Shah, and Zenati [29]	ML	ML through high tech operating rooms supports cognitive augmentation during surgical care. The future success of technological integration will be determined by how we handle data security and privacy.
Kim [30]	ML	Future aspects and advancements of ML are discussed in detail by the author, one of them being ML-based medical treatments to colorectal patients.
Ramesh et al. [31]	ANN	Studies on the future of ML demonstrated that ML algorithms like ANN are more effective than surgeons in detecting colorectal cancer.

## 4. Discussion

### 4.1. Application of ML in Colorectal Surgery

To optimize and decrease postoperative morbidity and mortality of patients, preoperative colorectal surgery patient assessment has become increasingly crucial. Various identification systems have been created in the past, but their clinical use is typically restricted. In medicine, AI is being examined for diagnostic and prognostic utility. This coincides with the growth of big data and the usage of electronic health records (EHR) in hospitals. AI can aid preoperative risk assessment by efficiently combining data such as basic demographics, biochemistry, and radiology results [1].

AI is frequently used in place of more precise words like ML or deep learning. So, for example, AI might be regarded as a parent area that includes subfields like ML, which includes techniques like neural networks and deep learning. Inspired by organic nervous systems, neural networks analyze data in layers of basic computing units similar to neurons. Deep neural networks (DNN) include more layers than basic 1-layer or 2-layer networks, allowing them to learn more complicated patterns than simple 1-layer or 2-layer networks. Computer vision (CV) and natural language processing (NLP) are two popular subfields of AI in medicine (and especially surgery). CV involves a system combining information from pixels, recognizing things inside images, and maybe even analyzing free areas within images. NLP allows robots to grasp human language as it is used in everyday life. It aims to grasp syntax and semantics to deduce meaning from phrases, sentences, or paragraphs. The primary sources in this review included necessary information on ML in colorectal surgery. The study by Hashimoto et al. [2], for instance, focused on the notion of ML and how various surgeons have adopted it in their practice. 

The article by Beyaz revealed the steps taken toward developing ML in surgery. Additionally, the review addressed the implementation challenges that we have encountered when trying to integrate ML, through the Da Vinci system, into their practice [3]. 

For instance, many surgeons have reported that they worry that the invention of better ML systems may lead to job losses for human surgeons. Authors like Kitaguchi et al. [7] and Wang et al. [8] evaluated the significance of ML applications in surgery; the former praised the convolutional neural network (CNN) technology for its accuracy. At the same time, the latter acknowledged the relevance of faster R-CNN ML in detecting colorectal cancer. Recently, the use of ML in colorectal surgery has gained much attention. A study conducted by Kitaguchi et al. [7], for example, revealed that the use of automated laparoscopic ML through a CNN to recognize surgical phases and actions automatically yielded an accuracy of 81% and 82%, respectively. This implies that many surgeons have adopted ML as it can give an accurate diagnosis. 

Similarly, Wang et al. [8] argued that ML-assisted pathological biopsy could be applied in the colorectal domain to examine colorectal illnesses and identify colon cancer. However, the integration of faster regions with CNN in diagnosing and treating colorectal cancer has not been systematically researched [8]. More importantly, a study carried out by Park et al. [9] aimed to develop an ML-based real-time analysis microperfusion model for indocyanine green angiography to forecast anastomotic complications during laparoscopic colorectal surgery and showed that ML had an accurate and consistent performance compared to traditional methods. 

DNN-based ML algorithms are also being used to detect colorectal polyps by defining the color channel in a different manner compared to conventional methods, generating better and more accurate results [32]. Another review by Mitsala et al. [10] indicated that CNN-based ML assists surgeons performing colorectal surgery to screen, diagnose, and treat colorectal cancer. For instance, we can use CNN to perform medical image analysis. According to Wang and Dong [11], automated polyp detection ML through conventional computer-assisted diagnosis (CAD) supports the diagnosis of the various stages of colorectal cancer. It does this by guiding us on the appropriate approaches to undertake. On a similar note, Merath et al. [12] proved that ML allows the creation of algorithms like the decision tree model that can accurately predict the possibility of patients developing problems after undergoing colorectal surgery. The study came to this conclusion after the decision tree algorithm accurately predicted the occurrence of complications in thirteen out of seventeen scenarios [12]. Yamashita et al. [13] applied ML in their practice by creating a deep learning model, referred to as MSINet, aiming to detect the prevalence of microsatellite instability (MSI) among individuals with colorectal cancer.

The study’s findings showed the approach’s effectiveness in predicting MSI, which exceeded the performance of gastrointestinal pathologists. Further, Echle et al. [14] developed a ShuffleNet deep learning system to clinically detect MSI in patients with colorectal tumors. The study results indicated that this system scored 65% on specificity and 95% on sensitivity in predicting MSI in colorectal cancer.

These findings suggest that ML can improve the procedures undertaken during colorectal cancer surgeries. Similarly, Ahmad et al. [15] claimed that the adoption of deep learning methodologies through CNN has increased in colorectal surgery, and they are now the most commonly used approach. Furthermore, many researchers and doctors have considered utilizing magnification imaging ML algorithms in practice [15].

Further evidence of the application of ML in colorectal surgery was obtained through the research initiated by Skrede et al. [16], which revealed that a prognostic marker was able to be established via deep learning to digitally scan tumor tissues stained with conventional hematoxylin and eosin. The tag demonstrated efficiency in separating stage I from stage II patients, which allowed surgeons to select adjuvant treatment, preventing therapy among low-risk individuals and recognizing the patients who would acquire maximum advantages from more intense medical programs [16]. These conclusions indicate that individuals are realizing and applying ML in the colorectal surgery domain.

The application of ML in colorectal surgery is also evident through the ML methodology established by Kudo et al. [17]. The authors developed an artificial neural network using information from 3134 patients with T1 colorectal cancer [17]. 

Further, Yuan et al. [18] established the ResNet3D + SVM classifier by using the ResNet-3D algorithm to detect synchronous peritoneal metastases in colorectal cancer. The researchers found the algorithm compelling, as it demonstrated an accuracy rate of 94% and a specificity degree of 93%. These results showed that the approach could be applied in colorectal surgeries to determine the patients needing additional surgery after endoscopic resection [18].

Another ML-based algorithm named EndoBRAIN was developed to identify colorectal neoplasms. This algorithm showed improved endoscopic activity. It was able to provide better and more significant results compared to 30 endoscopists collectively, including 10 experts [33].

The outcomes derived from these studies proved that ML is a suitable approach to enhancing the quality of care delivered to patients, especially colorectal cancer patients, before, during, and after surgery. All the studies outlined in this section prove that many colorectal surgeons have realized the significant role of ML through the various approaches applied to different situations in colorectal cancer care.

### 4.2. Advantages and Limitations of ML in Colorectal Surgery

The primary benefit of applying ML to colorectal cancer surgery is that it allows the prediction of the occurrence of lymph node metastasis, thereby reducing the need for surgeons to conduct additional surgeries after endoscopic resection of a T1 colorectal adenocarcinoma [19]. For instance, the ML algorithm model developed by Ichimasa et al. [19] permitted enhanced sensitivity and accuracy of algorithms used in this prediction. Another advantage of using ML in colorectal cancer is that it facilitates improved decision-making by ensuring the augmentation of the informed consent procedure and the decision to operate [20]. Further, ML-assisted electronic health records algorithms guarantee optimal surgical decision-making. This algorithm allows colorectal surgeons to recognize and mitigate potential risk factors of a process, derive appropriate decisions concerning postoperative management, and establish shared decisions relating to resource utilization [20]. Hildebrand et al. [21] claimed that ML-assisted MSI/dMRR tests through a CNN network are an effective tool for detecting MSI and predicting responses to immunotherapy, particularly among colorectal cancer patients. 

Another study by Luo et al. [22] presented the advantages of ML in colorectal surgery by proving that an ML-automated polyp detection system had the ability to increase the polyp detection rate and minimize the amount of time that surgeons spent in the process. This illustrates that the increased utilization of ML in colorectal surgery will reduce the workload for surgeons while simultaneously guaranteeing the efficiency of services.

Similarly, research spearheaded by Wang, Deng, and Wu [23] found that ML models that utilize magnetic resonance imaging (MRI) have proved effective in predicting the responses of various patients to chemotherapy, in addition to the evaluation of patient prognosis. The authors’ focus was patients with rectal cancer, and the algorithm yielded an accuracy rate of 95% [23].

This statement implies that the precise nature of ML-based algorithms has triggered their wide application in colorectal surgery. The different functions performed by various ML algorithms have supported their widespread use, from screening to surgery, which has made it easier for surgeons to undertake procedures that would take a long time to complete when using traditional approaches.

On the contrary, the main disadvantage of applying ML in colorectal cancer is its interpretability. Human scientists cannot evaluate how and why computers discern some patterns in various algorithms [24]. Another limitation of incorporating ML in colorectal surgery is that the majority of the ML systems require complex processing, which makes it challenging to extract data appropriately, thereby leading to erroneous results [24].

Furthermore, the application of ML in colorectal surgery faces the challenge of the expensive costs of offering training to staff on various deep learning networks. However, the main challenges facing the application of ML in colorectal surgery stem from the disconnection between scientific researchers and surgeons. This makes it challenging for developers to design ML systems that cater to the needs of patients and clinicians.

### 4.3. Future of ML in Colorectal Surgery

Another study by Hardy et al. insinuated that a promising future of ML algorithms, like deep CNN models, in colorectal cancer is possible given the current advancements in detecting the prevalence of colorectal cancer [25]. This implies that researchers will likely advance the technology used in detecting colorectal cancer to support effective treatment. The adoption of ML in colorectal surgery in the future has been supported in an article by Shung and Byrne [26], that posits that CNNs can enhance the quality of colonoscopy processes. This suggests that we are likely to implement the advantages of ML in colorectal surgery due to the benefits the algorithms provide during screening.

Recognizing these strengths will encourage further studies to develop the technology, to enhance the quality of other procedures involved in colorectal surgeries. Gao et al. offered a similar argument by demonstrating the success of a faster region-based CNN in diagnosing colorectal cancer. The technology enables surgeons to identify the prevalence of tumors and suggest appropriate medication for addressing the condition [27]. These advancements in technology will improve in the future, as surgeons will value strategies that make their work easier.

Moreover, ML is the future of colorectal surgery since we, as surgeons in different institutions, have the unique capability of driving innovations in the field instead of waiting passively for the technologies to advance [2]. The pivotal role that we have in promoting the adoption of ML implies that we will have to dedicate efforts toward developing the technologies since they make our work easier, improve efficiency, and enhance patient outcomes. According to McKendrick, Yang, and McLeod [28], ML in surgery will prompt mixed reality technologies, including advanced sensory systems, display schemes, and simulation platforms.

Additionally, the developers of future systems must engage in comprehensive research of those systems. Dias, Shah, and Zenati [29] reported that surgeons use ML algorithms through high-tech operation rooms to support surgical care via cognitive augmentation. This implies that the advantages attained by colorectal surgeons when applying ML will prompt them to develop the technologies further. Further, the successful integration of concepts in the future will largely depend on how the concerned parties handle issues of safety and confidentiality of data.

A study by Kim revealed that the future of colorectal surgery will involve instances where ML offers medical treatment to patients. Future medical technology discoveries will be based on intensive advances in nano and biotechnology, as well as on computers and the human genome. CAD, organ transplantation, gene therapy, tailored medications, and perhaps even age reversal would be achievable with this technology. 3D system technology will let surgeons visualize critical clinical aspects and plan out complex surgeries. Surgery might be performed by a medical robot under the supervision of surgeons in a virtual world. The incorporation of ML in colorectal surgery will probably yield positive results, thereby triggering more applications in the future [30].

ML algorithms have been tested in practically every medical sector. Other ML algorithm approaches such as decision tree systems, evolutionary computation, and hybrid intelligent systems have all been employed in diverse clinical situations. It has the potential to revolutionize medicine. More well-designed clinical studies are required before these new approaches may be used in the real world [31]. 

It seems as though the future application of ML is certain, provided that developers handle the current challenges facing different systems. Collaboration of developers, surgeons, and researchers will be necessary to achieve this goal, since they are able to envision the disadvantages of ML algorithms from different angles. It is essential for colorectal surgeons to rethink their work practices to integrate ML approaches, to permit the delivery of precise operations while minimizing the risk of harm to the patient.

Further, an interesting predictive model had been built by our team at the University of Basel and Clarunis for anastomosis leakage with a small population of patients having colon surgery as shown above. Our team utilized the “Streamlit” library of python to create a user-interactive prediction model “https://share.streamlit.io/aic-score-1/b” (accessed on 2 February 2022). 

This model has not yet been developed for clinical purposes and is still in a trial phase. It will be explained in the next publication, where we will present a glimpse of how it functions and obtains predictions.

### 4.4. Literature Gap

The primary literature gap realized via this review is the tendency of articles to focus on surgery in general when evaluating the application of ML in surgery. Very few publications have concentrated on ML in colorectal surgery. This suggests that surgeons in the field lack sufficient information to guide and enhance their practices, contributing to minimal application of ML in colorectal surgery.

The low application rates might imply that surgeons are not providing yet the quality care that patients would receive using ML algorithms. The lack of articles in the area further increases surgeons’ fears, especially about replacement by more intelligent systems. This could negatively affect the quality improvement of services given to patients, particularly during surgery. 

From this analysis, many studies have shown that the adoption of ML in colorectal surgery leads to reduced surgery time and lengths of hospital stay after surgery. Therefore, surgeons who refrain from adopting new technological approaches risk lagging behind and continuing to experience challenges, i.e., presented by open surgeries. The solution to this problem requires scientists to engage in comprehensive research on this topic. When participating in the studies, scientists should consider gathering the insights of surgeons to ensure that they present their views on the subject. This will make it easier to address the various concerns, ultimately promoting the integration of ML in surgery.

### 4.5. Future Work

The natural question arises of “When can this be implemented in the mainstream?” whenever a new and valuable technology emerges. In the case of ML and its application in medical fields, the questions include: “How feasible is it to include ML in the treatment process?”, “Is it cost-effective?”, and “Is it reliable and appropriate for technologies to diagnose and recommend treatment, or must humans be included?” These are a few natural considerations to establish some basic norms to future-proof the use of these tools. Surgeons’ collaboration with data scientists could pave the way for ML utilization in surgical domains [34]. 

Protective measures should be taken into consideration prior to proper implementation and before these ML methods become mainstream. The most immediate step should be to establish ML medical ethical guidelines. It will be useful to ascertain when it is and is not suitable to establish these technologies in the medical field. This will be an important step toward sustainable future applications.

In addition, there is a strong need to understand the underlying data from which conclusions have been drawn. One of the major problems faced by ML is the lack of credible data for the training of models or clear conclusions. For example, in studies conducted using CT scanned images, the total number of input images has ranged roughly from 5000 to 50,000 images, whereas the average number of images trained for the general purpose work of ML is in the hundreds of thousands. This can lead to unreliable results for difficult tasks, such as treating and diagnosing.

The issue of insufficient data will hopefully be addressed as ML prevails in the future. Big data facilitates better training and results of ML models. This involves a huge investment of money and time. Better arrangements need to be made that promote better data collection methods for enhanced decision making. As more institutions join these arrangements in the future, the amount of data accrued will increase. An open-source databank for the accessibility of information, such as symptomatology, geographic distribution, patient characteristics, imaging modeling, etc., could significantly benefit the researchers and scientists working in this domain.

### 4.6. Limitations

Many studies outlined in Table 1 tended to focus on the advantages of ML in colorectal surgery, while very few focused on the shortcomings. Some of the limitations are stated here. One of the main drawbacks of using AI in medicine is the fact that we are limited in data, which doubts the authenticity of the findings of its methods. Another limitation is the installation of AI equipment for its application, as not all can operate on such novice technology. Also, not all geographical locations or patients have similar medical datasets during their treatment, but AI tools are trained for universal data, thus making it not suitable for patient-specific treatments.

## 5. Conclusions

Many researchers have evaluated studies focusing on the future of ML in colorectal surgery. The purpose of this research was to demonstrate how surgeons have used ML in this medical field. The study results revealed that the adoption of ML in colorectal surgery in the future will lead to multiple benefits, such as enhanced efficiency. However, its success requires the development of cheaper systems, widespread availability, and collaboration between surgeons and relevant scientists and researchers. This will promote enhanced acceptance and the integration of ML features into colorectal surgery. In addition, it will equip surgeons with the proper knowledge, enabling surgeons to appreciate the advantages of ML, and refrain from viewing the concept as competition instead envisioning it as an intelligent assistant.

Furthermore, the results of this study indicate a lack of understanding of the challenges of ML, contributing to its slow adoption in colorectal surgery. The included articles have focused on the advantages of ML systems, and the lack of attention paid to the disadvantages of ML systems has contributed to their slow adaption. Thus, it remains vital for researchers in the field to conduct more studies on the topic to raise the public’s awareness of current and future issues facing the application of ML in colorectal surgery.

## Data Availability

The datasets used and/or analyzed during the current study are available from the corresponding author on reasonable request.
